# Phase Transformation in TiNi Nano-Wafers for Nanomechanical Devices with Shape Memory Effect

**DOI:** 10.3390/nano12071107

**Published:** 2022-03-28

**Authors:** Alexey Kartsev, Peter V. Lega, Andrey P. Orlov, Alexander I. Pavlov, Svetlana von Gratowski, Victor V. Koledov, Alexei S. Ilin

**Affiliations:** 1 Computing Center FEB RAS, 680063 Khabarovsk, Russia; a.kartsev@ccfebras.ru; 2 Bauman Moscow State Technical University, 105005 Moscow, Russia; alex.pav.2001@yandex.ru; 3 MIREA−Russian Technological University, 119454 Moscow, Russia; 4 Kotelnikov Institute of Radio Engineering and Electronics of the Russian Academy of Sciences, 125009 Moscow, Russia; andreyorlov@mail.ru (A.P.O.); victor_koledov@mail.ru (V.V.K.); alexey.ilin@phystech.edu (A.S.I.)

**Keywords:** nanomanipulation, nanomechanical tools, shape memory effect, size effects, TiNi

## Abstract

Recently, Ti-Ni based intermetallic alloys with shape memory effect (SME) have attracted much attention as promising functional materials for the development of record small nanomechanical tools, such as nanotweezers, for 3D manipulation of the real nano-objects. The problem of the fundamental restrictions on the minimal size of the nanomechanical device with SME for manipulation is connected with size effects which are observed in small samples of Ti-Ni based intermetallic alloys with thermoplastic structural phase transition from austenitic high symmetrical phase to low symmetrical martensitic phase. In the present work, by combining density functional theory and molecular dynamics modelling, austenite has been shown to be more stable than martensite in nanometer-sized TiNi wafers. In this case, the temperature of the martensitic transition asymptotically decreases with a decrease in the plate thickness h, and the complete suppression of the phase transition occurs for a plate with a thickness of 2 nm, which is in qualitative agreement with the experimental data. Moreover, the theoretical values obtained indicate the potential for even greater minimization of nanomechanical devices based on SME in TiNi.

## 1. Introduction

Already in the mid-1970s, the idea arose to use nano-objects and even large individual molecules as microelectronic devices [1]. But this idea flourished later in the era of nanotechnology when, in the 1990s, carbon nanotubes (CNTs) were synthesized and their outstanding functional properties were discovered [2,3]. Since then, various nano-objects [4], such as nanowires, were synthesized and a proof of concept of single devices was created on them. Both to create single nanodevices and to create microdevices and chips made from arrays of the aforementioned single nanodevices, nano-manipulation technologies are needed, with the help of which single nano-elements will be combined into individual nanodevices, then microdevices or chips from these nanodevices [5,6,7]. That is, there is a need for nano-manipulation technologies capable of providing nano-assembly and nano-manufacturing. Currently, there are many options for such technologies of nano-manipulation both on the plane [8] and in three-dimensional space [9,10]. In our opinion, one of the most promising is the method of mechanical spatial nano-assembling and nano-manufacturing, which can provide high-precision mechanical bottom-up nano-assembling and nano-manufacturing [10].

To carry out mechanical nano-manipulation and nano-assembly, it is necessary to carry out operations of mechanical capture and the transfer of nano-objects, such as nanowires and nanotubes and other elements and their assembly into unified systems. To carry out such operations, both a precise positioning system [11,12] and a nano-capture device with an end effector are required [13,14]. In this respect, composites of shape memory alloys (SMA) are very promising, which create giant reversible deformations up to 3–10% with a slight change in temperature (about 10 K), providing a very high strain-to-size ratio. It should also be noted their huge resource of the repetition cycles up to 10^6^, the absence of the need for training before use. All this creates a new promising trend in nano-manipulation-mechanical nano-manipulation based on nano-capturing tools based on SMA with thermoelastic martensitic transition. Moreover, it should be noted that the trend of nano-miniaturization requires a reduction in size, and the best mechanical nano-manipulation is provided by mechanical nano-tools, which have dimensions comparable to the manipulated objects. Therefore, the question of the fundamental limitation of the size of nano-instruments for mechanical nano-manipulation is naturally related to the minimum dimensions at which the corresponding phase transitions will occur. These studies are also of great value because they are expanding knowledge of the fundamental properties of the condensed state of matter and phase transitions to such an important area as the nanoscale.

The smallest thickness at which a phase transition (PT) can still occur is probably the thickness at which the material is already transformed into a two-dimensional state. It is from this point of view that the works [15] should be considered. When the size of a solid structure decreases, two effects arise: quantum confinement and surface effects. Quantum confinement is considered in the article [16]. Based on ab initio calculations, using the example of Sb (111) nanofilms, the authors of [16] demonstrate the interaction between two dominant effects, topological and electronic (topoelectronic). In this work, the features of transitions in thickness on a nanoscale are studied under the conditions of a wedge-shaped sample. Also, a number of other theoretical and experimental works are devoted to the study of phase transitions in wedge-shaped and conical specimens, for example [17,18].

Structural phase transition is a fundamental physical phenomenon that has been widely studied both theoretically and experimentally. According to Landau’s theory, in the case of second-order phase transitions, the coexistence of high-temperature and low-temperature phases is thermodynamically impossible in a bulk single crystal. Landau’s theory turned out to be very successful for bulk materials in second-order phase transitions, where surface effects can be neglected. However, when the material goes down to the nanoscale, the surface effects, as shown in many of the papers cited above, can no longer be neglected, therefore it is necessary to revise the behavior of phase transitions of second-order materials on the nanoscale, including the wedge shape [19]. Structural phase transitions and the shape memory effect were studied in [20,21,22,23] using molecular dynamics methods in a wide class of single crystals of pure metals and alloys at the nanoscale. Vivid effects of pseudoelasticity are predicted over a wide temperature range and very large reversible deformations.

An important problem in solid state physics is the features of martensitic phase transitions at the nanoscale [24,25,26] and corresponding size-dependence and limitations [27]. According to a phenomenological theory, the evolution of the order parameter in non-equilibrium systems is usually described by a generalized Ginzburg-Landau equation, where the critical size is related to the nucleus-forming process in first-order phase transitions and related critical nucleus size [28]. Experimentally for bulk polycrystalline alloys have proved a decrease of transition temperature for smaller grains, where the complete blocking of phase transition is observed for grains with linear size smaller than ~50 nm [29,30,31]. However, grains are 3D objects, and therefore, similar processes for low-dimensional objects such as nano-plates are of the most interest. For TiNi films the nonlinear declining character of the martensitic transformation temperature on their thickness is detected [32]. Along with experimental study, the computer simulations approaches have been applied to understand phase transitions at low-dimensional systems, specifically, the molecular dynamics (MD) method [33,34]. For example, according to Ref. [35], where the MD method was used to study the martensitic transformation temperature in the nanostructured Ti-Ni alloy-the SME degrades after several thermal cycles and load-unloading cycles [35]. However, we did not observe such a degradation effect for nano-wafers [36,37]; therefore, this issue is the subject to a more detailed study in the future.

The purpose of the present study is the theoretical and experimental study of the size effect at thermoelastic martensitic transition in wafer samples of TiNi intermetallic alloy with SME and discussion of the fundamental restriction of the minimal size of the nanomechanical devices, such as nanotweezers for 3D manipulation of the real nano-objects. The device presented in the paper was one of the main motivations to perform this study-simulations of nano-sized TiNi plates to find out limitations and optimal regimes for real application in nanoscience.

## 2. Methods

### 2.1. Experiment

In this work, we studied two types of samples obtained by ion etching from a thin TiNi film and a ribbon with a shape memory effect Ti_2_NiCu. For our studies the first sample was prepared in the form of a thin TiNi foil. The forward/reverse starting/ending temperatures in the bulk TiNi of the martensitic transformation are determined as *M*_s_ = 308 K, *M*_f_ = 298 K, *A*_s_ = 328 K, and *A*_f_ = 338 K respectively. A center of thin foil sample was thinned until a wedge-shaped hole emerged by usage of a GATAN Model 691 ion thinning machine. The crystal structure of the sample was studied on a JEM-2100 Transmission electron microscope (TEM) with a GATAN attachment for heating and cooling.

For the second experiment, the sample was made from a rapidly quenched Ti_2_NiCu alloy ribbon pseudo-plastically stretched by 1% by local etching and sputtering on FIB equipment. A small blank (~30 × 10 µm) was cut along the long edge of the ribbon, after which it was transferred to a holding grid for a transmission electron microscope (TEM). Then, at the focused ion beam (FIB) equipment, the sample on the grid was subjected to precision etching to give the required shape to the structure. By uniform etching on both sides, the ribbon was thinned to a thickness of several hundred nanometers by an ion beam directed parallel to the plane of the ribbon. At the next stage, composite bimetallic nano-actuators were formed, in which the SME layer acts as one layer, and the amorphous layer formed as a result of the redisposition of the removed material acts as the other layer. The process of formation of a new amorphous layer was carried out in a controlled way by sputtering a nearby crystalline layer using the precision nano etching mode with the sequential movement of the ion beam over a small area of the formed through a window from one side edge to the other, thus, on the opposite side, the material grew in the amorphous state. Next, the sample was again polished with a parallel beam of ions with reduced energy of several kilovolts on both sides to form a wedge shape at the tip of the actuators, to the thickness required for the TEM study. 

### 2.2. Computational Details

#### 2.2.1. Density Functional Theory

The total energy, electronic properties, and the structural optimization were performed by utilizing the Vienna Ab initio Simulation Package (VASP) [38]. Since the general form of the exchange-correlation functional is unknown, approximations are used. In this paper, the generalized gradient approximation (GGA) was used. For this method, there are various successful parameterizations that increase the accuracy of the approximation. In this work, the GGA-PBE parameterization (Perdew, Burke and Ernzerhof) [39] was applied. The electron-ion interactions were treated based on the augmented plane wave approach [40] and the pseudopotential method. The k-points grid with a maximum spacing of 0.1 Å^−1^ generated by the Monkhorst-Pack method was applied for integration in the irreducible part of the Bruluone zone of each TiNi phase and surface. The value of 500 eV was applied for cutoff energy of plane waves. To simulate a semi-infinite cell a vacuum region of 20 Å was included in the unit. The positions of all atoms were completely relaxed with convergence in forces of less than 0.05 eV/Å.

To calculate the surface energy in the TiNi alloy along the [001] direction, we considered two cuts in the Ni- and Ti-planes for the cubic phase, and four different cuts in Ti1, Ti2, Ni1 and Ni2 atomic planes. For the (110) direction, we considered TiNi-plane cuts both in the cubic and in the monoclinic phases. Along the (111) direction, we utilized two different cuts for the cubic phase — in the Ti- and Ni-plane. And for a monoclinic cell we considered only one cut—a cut along the TiNi-plane. Along the direction [122], one cut was used for the cubic phase along the TiNi-plane, and for the monoclinic phase four different cuts—2 cuts in Ti1-, Ti2- an and 2 cuts in Ni1-, Ni2-planes. 

To simulate a semi-infinite bulk, we utilized a unit cell containing six equiatomic layers, where three layers are fixed mimicking the effect of the bulk material. In the next three layers, the positions of atoms were relaxed, thereby recreating the effect of a surface restructuring. The formula for calculating surface energy for TiNi:

(1) $$E^{surface}=\frac{1}{2A}\left[E_{slab}^{total}-\frac{1}{2}\;E_{TiNi}^{bulk}-E_{Ni}^{bulk}\left(N_{Ni}-N_{Ti}\right)\right]$$

where $E_{slab}^{total}$ and $E_{Ni}^{bulk}$ is the total energy per formula unit of semi-infinite and bulk materials respectively. *N*_Y_ and *A* are the number of Y atoms (Y = Ti, Ni) in the utilized unit cell and the size of the surface in the modeled cell correspondingly.

#### 2.2.2. Molecular Dynamics Method

The MD simulation was carried out on the basis of a modified embedded atom method for second nearest neighbors with an interatomic potential, specially developed for the TiNi system [41]. This potential was developed to accurately reproduce the phase transformation induced by temperature or mechanical stress in an equiatomic TiNi alloy. This potential reproduces the phase transformation from the B2 austenite phase to the B19’ martensitic phase, as well as the fundamental physical properties (structural and thermodynamic) of the corresponding intermetallic compounds. All calculations were performed with a cutoff radius of 5.0 Å, which is greater than the distance to the second nearest neighbor in the equilibrium B2 structure of the TiNi alloy.

Then a series of MD simulations were carried out using the LAMMPS code [42] with a time step of 2 fs. The system was brought to equilibrium at a temperature of 500 K using a Nose-Hoover thermostat [43,44] and a Parrinello-Rahman barostat [45]. Periodic boundary conditions were applied in two dimensions, while an artificial empty space was introduced along the third direction (*z*-axis) to simulate a vacuum. During the simulation, the longitudinal dimensions of the cell, the angles of the cells, and the positions of individual atoms were completely relaxed. On the originally generated cells, the process of energy minimization over the positions of the atoms was applied by the conjugate gradient method.

The phase transformation during cooling of the sample was investigated by carrying out molecular dynamics simulations in an isobaric medium at external atmospheric pressure. From an initial temperature of 500 K, the temperature gradually decreased to 25 K at a cooling rate of 0.5 K/ps. Every 100 steps, the parameters of the simulated cell and the coordinates of the atoms were recorded for the analysis and observation of phase transformations.

The construction and visual analysis of the phase composition was carried out by the Voronoi polynomial method [46], implemented in the OVITO software package [47]. Directly at the grain boundaries and plate surfaces, a small part of the atomic structure was not recognized as a result of the influence of surface effects and permanent deformations. In the figures, the atoms shown in blue represent the B2 austenite phase and the B19’ martensite phase in red.

## 3. Results and Discussion

### 3.1. Experimental Part

#### 3.1.1. Martensite Transformation

**Figure 1:**
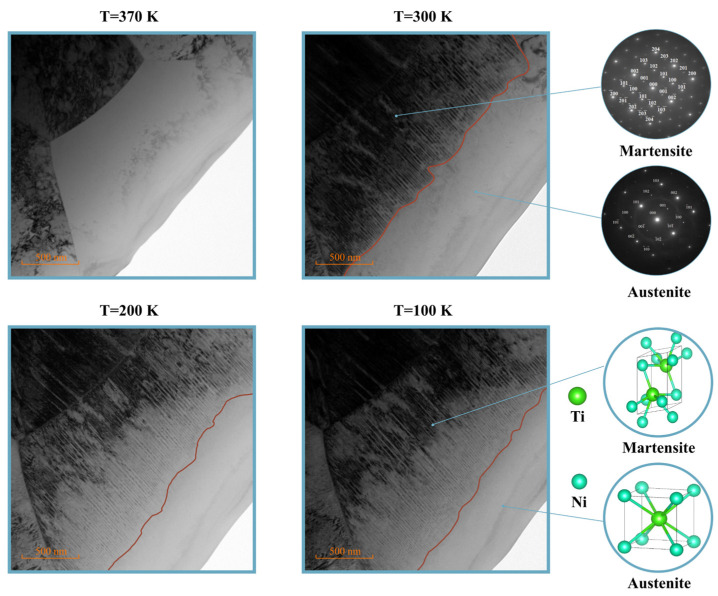
TEM image of a wedge-shaped TiNi sample with decaying thicknesses (from middle to edge) at room temperature. The boundary of the martensite-austenite transition with retained austenite at the edge is clearly visible in the TEM image of a wedge-shaped TiNi sample at different temperatures. Microdiffraction images and corresponding crystal structures are shown in the inserts.

**Figure 2:**
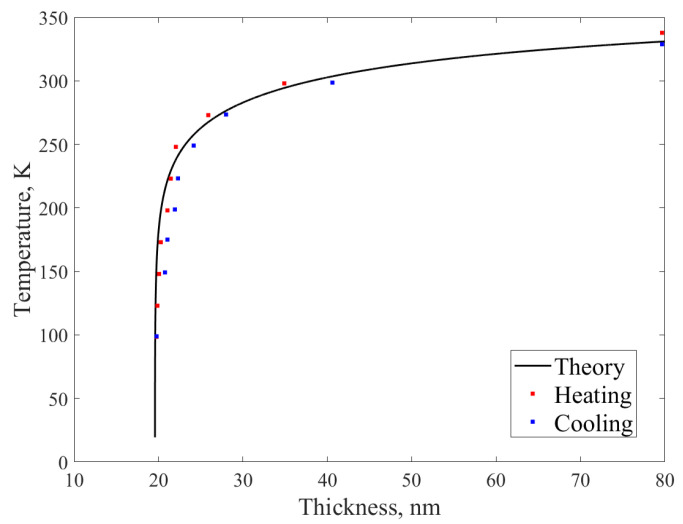
The Measured TiNi-wedge thickness at the transition boundary at different temperatures upon heating/cooling. $T\left(h\right)\;≂\;T_{C}/\left(1-A\cdot ln\left[\frac{B}{C+D/h}-1\right]\right)$ curve approximation according to Ref. [49], where *A, B, C, D* are free parameters and *T_C_* ≃ 330 K (phase transition temperature for bulk TiNi).

The TEM method was used to study the crystal structure of individual cross-sections of the TiNi thin plates in the 40–450 K temperature range using a specific technique (see details Ref. [48]). In Figure 1 are shown the micro images obtained using TEM in the area near the sample edge where the martensite-austenite border is clearly visible and approaches the edge with decreasing temperature-moves to the region of minimum plate thickness. And eventually the boundary gets stuck-the transition is blocked and does not occur even at lower temperature. Measuring wedge thickness h at the martensitic-austenitic boundary with a small step in temperature *T*, a *T(h)* dependence plotted at Figure 2. The thickness measurement for the plate was performed on a TEM setup by utilizing electron characteristic energy loss spectroscopy (EELS). The *T(h)* curve obtained here for the TiNi plates has a similar character dependence in the case of Ti_2_NiCu alloy [48,49].

#### 3.1.2. SME-Based Nano-Device in TEM

**Figure 3:**
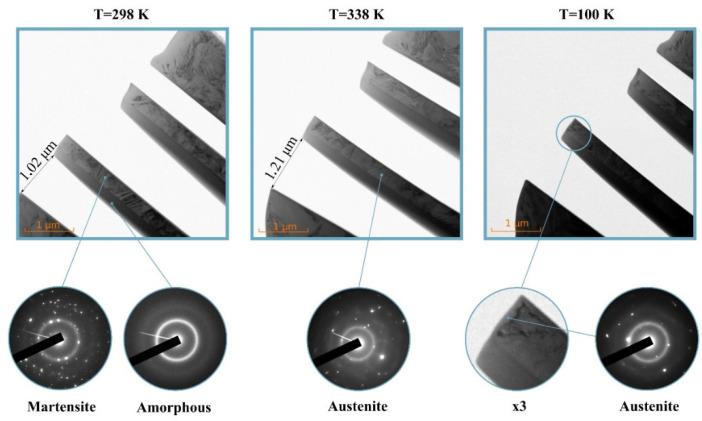
Structure and shape of nano actuators in TEM (*in-situ*) at different temperatures during thermal cycling.

The Ti_2_NiCu nano-actuators obtained by the original method [27] were studied in TEM, where they demonstrate reversible giant deformations during heating-cooling cycles. In the experiment, the mechanical movement (deflection) of the fabricated nano-actuators due to temperature changes was clearly recorded, this small relative displacement is shown in photographs taken at two extreme temperatures, below and above the thermoelastic phase transition of the alloy (see Figure 3). 

The alloy that has been employed for the nanoactuator device fabrication (Ti_2_NiCu) is different from the one been considered for MD simulations and wedge-shaped samples (pure TiNi). However, Cu-dopping is just a technological method in order to make device fabrication easier. This kind of doping does not affect the final SME properties. However, Ti_2_NiCu in contrast to pure TiNi have transformation temperatures near the room temperature value with a smaller width of SME hysteresis (~1 K). This alloy device operates at room and slightly higher temperatures without a drift. Overall, Ti_2_NiCu-based devices are more controllable, cheaper for mass devices production, and more suitable for real technological applications [50].

In accordance with Reference [51], the relative deformation of the actuator $\varepsilon =\frac{8h\lambda }{3L^{2}}$, where λ is the deflection, *h* is the thickness of the TiNi layer. Here for samples prepared we have observed *ε* = 0.7% and *λ* = 200 nm. Microdiffraction patterns (see insets in Figure 3) prove the coexistence of martensite and austenite phases in the nanolayer at low temperature. While upon heating the martensitic twins disappear (see Figure 3) and the nanolayer transforms to an austenitic state. The thickness of the nano-actuators was measured by EELS and there was 30 nm at the tip of the actuator.

### 3.2. Computer Simulations

#### 3.2.1. Thermodynamic Approach Based on the DFT Method

From a classical thermodynamic point of view, the observed dependence can be explained as a balance between different energetic contributions to the Gibbs energy. Considering the stable state of the system, the following expression can be written [52] for the transition from austenite to martensite as a function of temperature *T* and TiNi wafer thickness *h*:

(2) $$G^{A\to M}\left(h,T\right)=\Delta G^{bulk}+\Delta E^{surface}+\Delta E^{interface},$$

where $\Delta G^{bulk}$–the free energies difference between bulk martensite and austenite phases, $\Delta E^{surface}$– the difference in surface energies, $\Delta E^{interface}$–the energy of the new phase boundaries creation. 

$\Delta G^{bulk}$ is the main driving force of martensitic transformation in a bulk material, in nano-plates, a comparable contribution to $G^{A\to M}$ will be given by the $\Delta E^{surface}$ and $\Delta E^{interface}$ terms. Using the DFT method, the surface energies for TiNi were calculated for both cubic and monoclinic systems for the main crystallographic directions (001), (100), (111), (122). According to DFT the (001) surface in the austenite phase with a cut along with Ni and with a formation energy of 1.245 J/m^2^ turned out to be energetically more favorable among other considered crystallographic directions/cuts. While the total energy and (001)-surface energy difference between two phases are found to be $\Delta G^{bulk}=$ −8.971Δ10^−21^ J/f.u. and $\Delta E^{surface}$ = 0.287 J/m^2^ respectively. These values give evidence that surface energy drives the systems in the direction of the austenite stabilization, while the martensite phase is energetically more preferable stable for bulk phase. Hence, due to the surface effects, austenite may be retained as the most energetically favorable phase. In contrast to the above explanation, classical theory based on the Ginzburg-Landau theory failed to explain that [48]. On other hand, offered thermodynamic description is consistent with a more advanced phenomenological model built in the frameworks of the dislocation-kinetic theory [53].

#### 3.2.2. Molecular Dynamics Results

From an atomistic point of view, we are faced with the task of a theoretical quantitative description of systems containing hundreds of thousands to several million atoms. At this stage in the development of science and technology, the posed multi-particle problem of the numerical description of the experimental results obtained can be correctly solved only by the modern method of molecular dynamics based on the interatomic potential obtained by machine learning [41]. While the methods outlined above give only a qualitative description consistent with the experimental results. And the phenomenological theory developed to describe such systems [49,53] operates with a variety of “adjustable” parameters. Moreover, a key aspect of describing a phase transition within the framework of the molecular dynamics method is the creation of a correct interatomic potential, through which the transition from a quantum-mechanical description of a system of atoms to a classical one takes place.

Within the groundwork of classical molecular dynamics, atoms are seen as point objects interacting via the interatomic potential. In our work this interatomic potential is constructed using a machine learning method based on data obtained from ab initio calculations [54]. Thus, the numerical problem is greatly simplified-instead of solving the Schrödinger equation for a huge number of quantum particles (ions and electrons), it is necessary to solve the problem of the interaction of classical particles (atoms) with each other. Taking more atoms into account leads to a more accurate solution, but demands a higher computational cost-the usual solution here is to limit all interactions between atoms to the nearest neighbors or at some distance from each atom (the so-called “cutoff radius”).

**Figure 4:**
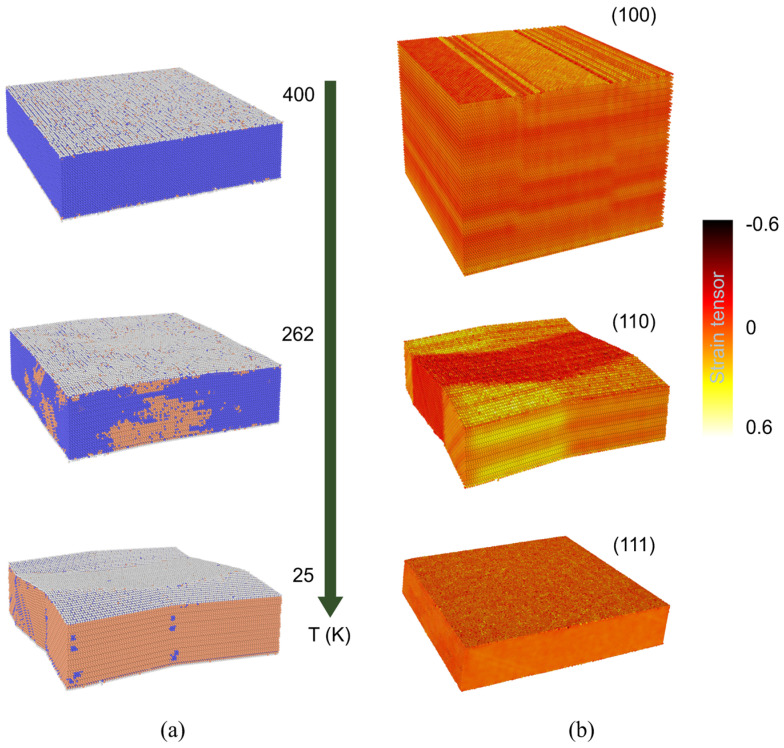
(**a**) The martensitic transition of the TiNi wafers through (110) direction and thickness is 25 nm. Blue color corresponds to the austenite and orange color to martensite. (**b**) The strain tensor (sum of non-diagonal elements) of the TiNi wafers through (100), (110), and (111) directions.

**Figure 5:**
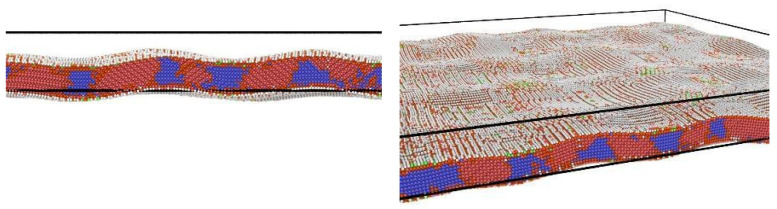
The formation of the TiNi martensite phase under cooling (down to T ⋍ 40 K) using the molecular dynamics method within the LAMMPS package. The rectangular simulation box shown is considered in the frameworks of the periodic boundary conditions applied in the x-y plane, and finite boundary conditions utilized along the *z*-axis. Number of atoms in the system is 0.3 × 10^6^ atoms. The blue color denotes austenite (B2), the red color corresponds to the martensite phase (B19’), and the white-green color corresponds to the atoms located on the surface and does not correspond to any of the bulk phases.

Martensite phase formation shows at Figure 4a during the cooling of the (110)-terminated plate sample with 25 nm thickness. Martensite at low temperatures is maintained at the entire sample. Growth of the martensite starts almost instantaneously on the time-scale used to simulate the cooling process (the utilized time step much bigger than the characteristic time of martensite growth related to the sound velocity). The modeling results for the sample with 25 nm thickness are different from the results obtained for a plate with a thickness ~5 nm (see Figure 5). Moreover, our results show the presence (about 70%) of a residual austenite at low temperatures in the thinnest plate (see Figure 5). This feature is a key difference from the process of martensite formation in a 25 nm thick TiNi wafer. 

To characterize shape mutation, we have calculated the strain distribution of the TiNi wafers corresponding to martensitic transformation. The sum of non-diagonal elements of the strain tensor $\sum_{i\neq j}^{}\varepsilon_{ij}$ for (100)-, (110)- and (111)-terminated plates at low temperature (~25 K) are shown in Figure 4b. It can be seen that there is elastic energy spent on the twins’ formation for (100)- and (110)-terminated plates. While no twins were observed for the (111)-terminated $\varepsilon_{ii}$ are playing a crucial role similar to the common thermal expansion [55]. Since twins with {111} habit planes have relatively small energy barriers compared to the other twins in TiNi [56] their formation is preferable. And in the case of a thin plate with (111)-termination can lead to the situation where the whole plate with a small thickness acts as a single twin. Therefore, it is fair to expect the absence of SME for such structures due to the absence of twins. 

**Figure 6:**
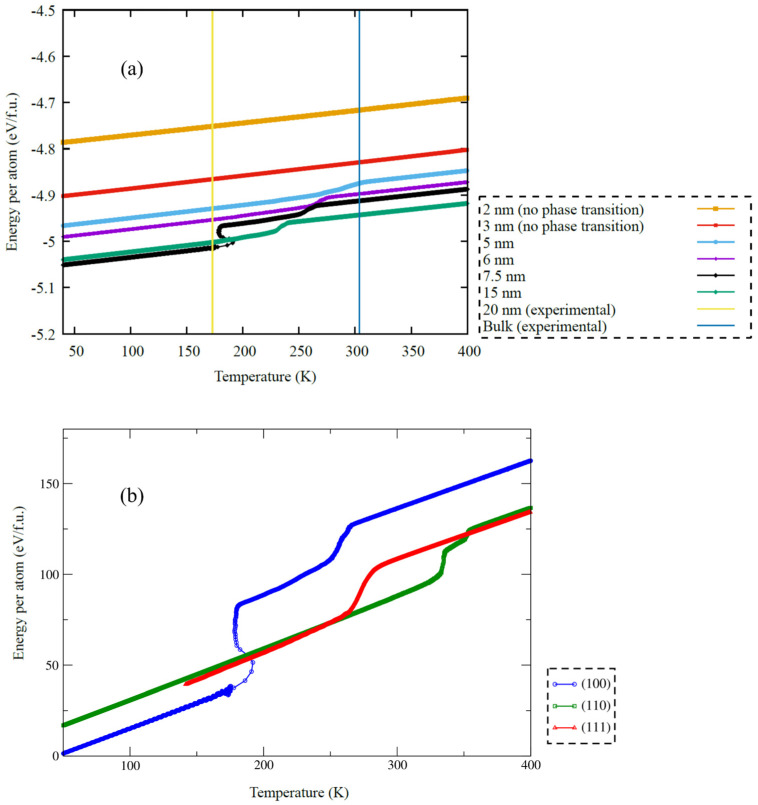
(**a**) Temperature versus total energy per atom for 10 nm and 2 nm thicknesses of a TiNi wafer by the molecular dynamics method. Dependence of the total energy per formula unit on temperature during cooling of TiNi plates with thickness values h ≈ 25÷35 nm slightly higher than the critical value $h_{cr}$. (**b**) Blue, green, and red lines correspond to plates with different sections along the crystallographic planes (100), (110), and (111), respectively.

At a plate thickness *h_c_*_r_ ~ 2 nm (Figure 6a), the obtained total energy results clearly indicate a complete blocking of the formation of the martensite phase, which is in qualitative agreement with the observed experimental results, dislocation-kinetic model [48,49] and thermodynamic estimations for the surface-bulk energies balance $\Delta E^{surface}/\Delta G^{bulk}$. However, the obtained results of the numerical experiment quantitatively differ from the experimental ones by an order of magnitude. We attribute this discrepancy to the presence of various types of defects in a real experiment (including implanted gallium ions and an amorphous surface) and the presence of a finite cooling rate.

Figure 6b shows the dependence of the total energy of the simulated plates with different crystallographic orientations of the surfaces. The effect of the orientation of the cross-section on the phase transition temperature in thin TiNi wafers was shown for the first time. This behavior can be explained as an increase in the specific contribution of surface energy $\Delta E^{surface}$ to free energy $G^{A\to M}\left(h,T\right)$ with decreasing plate thickness, which indicates the critical role of surface effects in describing martensitic transformations in thin TiNi wafers and austenite stability.

## 4. Conclusions

Bringing together methods of classical molecular dynamics, DFT simulations and experimental study, a description of the martensitic phase transition in TiNi alloy is investigated for bulk samples and nanoscale plates. Theoretical estimations, computer simulations, and experimental results show the complete blocking of the martensitic transition at the nanometric scale for TiNi plates. Additionally, it has been shown that the crystallographic orientation of the thin TiNi plate surface has a significant effect on the martensite phase formation and can lead to the absence of SME. This study allowed us to gain a clearer understanding of the martensitic processes occurring in Ti-Ni nano-wafers and grasp factors affecting their physics. This allowed us to fabricate the “comb shape” nanodevice where the active SME elements with a record thickness were utilized. We have shown that this nanoscaled nanoactuator can operate freely based on the reversible deformation effect due to the martensitic phase transition. This opens up bright perspectives for decreasing the actuator sizes based on the TiNi alloys where the size of the tools is comparable with a manipulatable object at the nanoscale. 

## Data Availability

The data presented in this study are available on request.
